# Frequency and Characteristics of Craniomaxillofacial Tumors: A Five-Year Retrospective Institutional Study

**DOI:** 10.3390/jcm14176256

**Published:** 2025-09-04

**Authors:** George-Dumitru Constantin, Ioana Veja, Serban Talpos Niculescu, Crisanta-Alina Mazilescu, Teodora Hoinoiu, Valentina Oana Buda, Roxana Oancea

**Affiliations:** 1 Discipline of Clinical Practical Skills, Department I Nursing, Faculty of Medicine, “Victor Babeș” University of Medicine and Pharmacy, 300041 Timișoara, Romania; george.constantin@umft.ro (G.-D.C.); tstoichitoiu@umft.ro (T.H.); 2 Department of Dental Medicine, Faculty of Dentistry, “Vasile Goldiș” Western University of Arad, 310025 Arad, Romania; veja.ioana@uvvg.ro; 3 Faculty of Dentistry, Department of Oral and Maxillofacial Surgery, “Victor Babeș” University of Medicine and Pharmacy, 2 Eftimie Murgu Sq., 300041 Timișoara, Romania; 4 Teacher Training Department, Politehnica University Timisoara, 300596 Timisoara, Romania; 5 Faculty of Pharmacy, “Victor Babeș” University of Medicine and Pharmacy, 2 Eftimie Murgu Street, 300041 Timisoara, Romania; buda.valentina@umft.ro; 6 Research Center for Pharmaco-Toxicological Evaluation, “Victor Babeș” University of Medicine and Pharmacy, 2 Eftimie Murgu Square, 300041 Timisoara, Romania; 7 Research and Processing Center for Medicinal and Aromatic Plants, “Victor Babeș” University of Medicine and Pharmacy, E. Murgu Sq., No. 2, 300041 Timisoara, Romania; 8 Translational and Experimental Clinical Research Centre in Oral Health, Department of Preventive, Community Dentistry and Oral Health, Faculty of Dental Medicine, “Victor Babeș” University of Medicine and Pharmacy Timisoara, 300041 Timisoara, Romania; roancea@umft.ro

**Keywords:** craniomaxillofacial neoplasms, head and neck neoplasms, oral cavity cancer, salivary gland neoplasms, oropharyngeal neoplasms, hypopharyngeal neoplasms, paranasal sinus neoplasms, hospital-based study, Romania, ICD-10 recoding

## Abstract

**Background:** Hospital-based data can complement registry estimates for cranio-maxillofacial (CMF) oncology, particularly in under-reported regions. We aimed to describe the institutional case-mix of CMF tumor diagnoses, standardized to ICD-10 sites, and to quantify trends using visit-normalized indicators. **Methods:** We conducted a retrospective, observational, single-center, hospital-based study of diagnosis-level encounters (2012–2016). Diagnoses were recoded to ICD-10 and restricted to CMF sites (lip, oral cavity, major salivary glands, oropharynx/hypopharynx, nasal cavity/middle ear, paranasal sinuses, eye/adnexa). The primary indicator uses a strict CMF set (malignant CMF codes plus D00.0 and D14.1); odontogenic cysts and non-neoplastic jaw lesions (K09–K10) were excluded, while benign CMF neoplasms are reported descriptively for site distributions. **Results:** We identified 2729 malignant CMF diagnoses over 2012–2016, peaking in 2014 (*n* = 751) and lowest in 2016 (*n* = 367). The combined malignant rate (per 1000 total visits) was 30.6, 43.9, 52.6, 34.4, and 26.7 for 2012→2016. The proportion of malignancies within the strict CMF set was 99.2%, 97.3%, 97.9%, 96.8%, and 95.1%, respectively (overall 97.4%). The most frequent malignant sites cumulatively were the palate (*n* = 416), parotid gland (*n* = 376), floor of mouth (*n* = 344), gingiva (*n* = 282), and mouth, unspecified (*n* = 179). **Conclusions:** After ICD-10 recoding and restriction to CMF sites, malignant tumors predominated within the institutional, diagnosis-level case-mix, with a 2014 peak followed by a decline. These indicators are case-mix monitors and not population incidences; interpretation should consider coding practices and service-mix changes across years.

## 1. Introduction

Cancer remains a major public health concern worldwide, and malignancies arising in the head and neck contribute substantially to morbidity, mortality, and healthcare utilization [1,2]. Within this group, tumors of the craniomaxillofacial (CMF) region—here defined as neoplasms of the lip, oral cavity, major salivary glands, oropharynx/hypopharynx, nasal cavity and middle ear, paranasal sinuses, eye and adnexa, and craniofacial bones—pose distinctive diagnostic and therapeutic challenges. These lesions threaten essential functions such as speech, mastication, and deglutition, and they affect facial appearance and psychosocial well-being, necessitating specialized, multidisciplinary care pathways [3,4,5].

The etiologic landscape across CMF sites is heterogeneous. Tobacco and alcohol synergistically drive risk for many oral cavity and oropharyngeal cancers, while oncogenic human papillomavirus (HPV) has reshaped the epidemiology and prognosis of oropharyngeal disease in numerous settings [6,7,8]. Additional contributors—ultraviolet exposure for lip cancer, occupational or environmental exposures (e.g., wood dust) for sinonasal tumors, chronic inflammation, and oral health disparities—vary across populations and over time [9,10,11]. Despite advances in surgery, radiotherapy, and systemic therapy, outcomes remain strongly stage-dependent, and delays in presentation and diagnosis continue to erode survival for a substantial subset of patients [12,13].

Robust, site-specific surveillance is essential for prevention, early diagnosis, and service planning. However, in parts of Eastern Europe, including Romania, reporting for CMF tumors is limited or inconsistently aggregated into broader “head and neck” categories that may include non-CMF sites (e.g., larynx), hindering actionable comparisons [14,15]. Variations in coding practices, incomplete topographic specification, and mixed data sources further complicate the interpretation of temporal trends and regional differences, including potential gaps between urban and rural populations in access to timely diagnosis and specialist care [16,17,18].

Hospital-based studies can complement population registries by describing the case mix, care pathways, and surgical workload observed in referral centers. Yet, comparability requires standardized case definitions and transparent denominators, with a clear distinction between caseload (diagnosis-level counts) and incidence (population-based rates), and consistent site coding across years. Prior institutional reports from the region have variably grouped CMF with non-CMF head and neck sites or relied on heterogeneous coding systems, limiting cross-study synthesis and utility for service planning [19,20,21].

To address these gaps, we analyzed CMF tumor diagnoses over a five-year period (2012–2016) at a tertiary referral department serving multiple counties in Western Romania. This study describes the frequency and distribution of CMF diagnoses and related oncologic procedures and quantifies temporal patterns using institution-level rate metrics aligned with clinical activity. By applying standardized, site-specific categorization, we aimed to provide evidence that can inform local pathways and contribute to more consistent CMF reporting.

This study evaluates CMF neoplasms at the diagnosis level in a hospital-based cohort, restricted to ICD-10 CMF sites (lip, oral cavity, major salivary glands, oropharynx/hypopharynx, nasal cavity/middle ear, paranasal sinuses, eye/adnexa, craniofacial bones, and head/neck skin); non-neoplastic odontogenic/jaw lesions (K09–K10) are excluded, and selected benign CMF neoplasms are summarized descriptively for site distributions.

Our primary indicator focuses on malignant CMF neoplasms, using a strict CMF denominator defined a priori as malignant CMF codes plus D00.0 (oral cavity carcinoma in situ) and D14.1 (benign middle ear), with trends reported as a combined malignant rate per 1000 total visits (outpatient + ED)—an institutional case-mix metric rather than a population incidence.

## 2. Materials and Methods

### 2.1. Study Design and Setting

We conducted a retrospective, cross-sectional, single-center, hospital-based study. The study period was 1 January 2012 to 31 December 2016. The department delivers comprehensive CMF care across outpatient clinics, the emergency department (ED), urgent outpatient pathways, and inpatient services, including diagnostic work-up, biopsies, tumor excisions, and major resections with or without reconstruction.

### 2.2. Data Sources and Eligibility

Data Source and Period. We queried the hospital’s integrated EHR (InfoWorld Connecting Healthcare) for encounters managed by CMF services between 1 January 2012 and 31 December 2016 across outpatient clinics, emergency department (ED), urgent outpatient, and inpatient streams.

Initial Retrieval. We retrieved all records carrying at least one ICD-10 code from neoplasm chapters C00–C97 or D00–D49 and/or matching neoplasm-related terms in diagnosis text fields.

Final Inclusion (Case Ascertainment). Encounters were included if at least one diagnosis mapped to CMF topographies (lip, oral cavity, major salivary glands, oropharynx/hypopharynx, nasal cavity/middle ear, paranasal sinuses, eye/adnexa, craniofacial bones, skin of face/scalp/neck restricted to head/neck). Inclusion required explicit head/face/neck documentation when ICD-10 categories could span multiple sites.

Exclusions. We excluded the following: (i) non-CMF or ambiguous topographies (e.g., C32 larynx, C71 brain, C79.x distant metastases outside head/neck, C80 unspecified primary), (ii) unspecified benign/uncertain behavior codes without CMF site resolution (D36.x, D48.x), and (iii) non-neoplastic odontogenic/jaw lesions (K09–K10; cysts, inflammatory, and other non-tumoral conditions). ED encounters were included only if formally registered under CMF.

Unit of Analysis. The analysis is diagnosis-level: each coded tumor diagnosis linked to an encounter counts as one record; encounters with multiple tumor diagnoses contribute to multiple diagnosis-level records.

Rate Scope. For visit-normalized indicators, numerators exclude inpatient diagnoses to match the outpatient+ED denominator.

### 2.3. Case Definitions, Coding, and Unit of Analysis

Case definitions and coding. All diagnoses were recoded to ICD-10 and restricted to CMF topographies. Included malignant CMF codes were as follows: C00–C14 (lip, oral cavity, oro-/hypopharynx), C30–C31 (nasal cavity, paranasal sinuses), C41.0 (bones of skull and face), C43.3–C43.4 and C44.3–C44.4 (skin of face/scalp/neck), C47.0 and C49.0 (peripheral nerve and soft tissue of head/face/neck), C69 (eye/adnexa), and C77.0 (regional lymph nodes of head/face/neck). For the strict CMF analytic set used in the primary indicator, we retained malignant CMF plus D00.0 (oral cavity CIS) and D14.1 (benign middle ear). For descriptive site distributions, we additionally summarized selected benign CMF neoplasms (e.g., D10.x benign mouth/pharynx; D16.4 benign bones of skull/face; D17.0 lipomatous skin/subcutis of head/face/neck; D18.x hemangioma/lymphangioma at head/neck sites; D23.3–D23.4 benign skin of face/scalp/neck; and D03.3–D03.4/D04.3–D04.4 in situ melanoma/skin of face/scalp/neck). Excluded were non-CMF or ambiguous codes (e.g., C71 brain, C79.x distant metastases outside head/neck, C80, D36.x/D48.x unspecified sites), laryngeal sites, and non-neoplastic odontogenic/jaw lesions (K09–K10). Where ICD-10 categories could map to multiple topographies, inclusion required explicit head/face/neck documentation in the EHR.

We report clinically relevant classes (ICD-10 site proxies):

1. Oral/oropharyngeal (C00–C14; SCC-predominant sites);
2. Salivary glands (C07–C08);
3. Cutaneous head/neck (C43.3–C43.4, C44.3–C44.4; in situ D03.3–D03.4, D04.3–D04.4);
4. Sinonasal/middle ear (C30–C31; D14.1 benign middle ear);
5. Eye/adnexa (C69);
6. Craniofacial bone/soft tissue/peripheral nerve (head/neck) (C41.0, C49.0, C47.0; selected benign D16.4/D17.0/D18.x);
7. Regional nodes/lymphoid presentations (C77.0; C82/C83 head–neck).
  Note: ICD-10 does not encode morphology; therefore, SCC cannot be confirmed at the record level. Odontogenic tumors cannot be isolated cleanly and are reported within the craniofacial bone/soft-tissue/nerve class.

Histopathology. Where available, histopathology (biopsy or resection) was linked at the encounter level—from the same visit or within 30 days of the index encounter. The histopathology-confirmed subset is presented descriptively and does not overwrite coded diagnoses.

### 2.4. Variables and Measures

From each eligible encounter, we extracted the following: sex; age at visit (<18, 18–39, 40–64, ≥65 years); residence (urban/rural per National Institute of Statistics definitions current at the time of care); county of residence; date and type of encounter (outpatient, ED, urgent outpatient, inpatient); primary and secondary diagnosis codes; procedure labels (e.g., biopsy, skin/lesion excision, mandibular/maxillary resection, vascular ligation); and histopathology availability (yes/no).

Clinical Denominators and Rate Metric. To contextualize diagnosis counts, we used annual volumes of outpatient and emergency department (ED) activity. The primary rate reported is a combined institution-level indicator: malignant CMF diagnoses per 1000 total visits (outpatient+ED) in the same year. Numerators exclude inpatient diagnoses to match the outpatient+ED denominator. This metric is descriptive and reported without confidence intervals.

### 2.5. Outcomes

Primary Outcomes. (i) Annual counts of CMF tumor diagnoses overall and by ICD-10 CMF site category; (ii) the proportion malignant within the strict CMF analytic set (diagnosis-level), defined a priori as malignant CMF diagnoses plus D00.0 and D14.1; and (iii) the combined malignant CMF rate per 1000 total visits (outpatient + ED), with inpatient diagnoses excluded from the numerator.

Secondary Outcomes. Setting mix (outpatient/ED/inpatient), demographics, referral geography, and procedure frequencies (linked to the same encounter as the diagnosis).

### 2.6. Data Management and Quality Assurance

Raw EHR extracts were de-identified and imported into an analysis dataset. Standardized cleaning included removal of administrative duplicates at the encounter-ID level; normalization of dates; harmonization of diagnosis text to ICD-10 codes; and anatomic plausibility checks (e.g., ensuring malignant skin neoplasms mapped to head/face/neck when included). If a single encounter carried multiple tumor codes, each diagnosis was retained as a separate record linked to its encounter. Procedure categories were derived from hospital operating room logs and procedure labels, mapped via a predefined dictionary; counts reflect procedures linked to the same encounter as the tumor diagnosis.

### 2.7. Handling of Missing Data

We quantified missingness for key variables (age group, residence, county, histopathology) and report relevant denominators alongside all percentages in tables and figures. Missing data were not imputed; available-case denominators are used and explicitly stated.

### 2.8. Statistical Analysis

Analyses were performed using IBM SPSS Statistics v25 and were descriptive. Categorical variables are summarized as counts and proportions. The combined malignant CMF rate is reported per 1000 total visits (outpatient+ED) without inferential statistics. No sample size calculation or formal hypothesis testing was prespecified. Year-to-year variability and setting mix were examined to contextualize fluctuations in malignant proportions and rates.

### 2.9. Reporting Standards and Ethics

This study was conducted in accordance with the Declaration of Helsinki. Ethical approval for this retrospective analysis of de-identified records was obtained from the Victor Babeș University of Medicine and Pharmacy Ethics Committee, Timișoara, Romania (No. 11 approved 8 January 2025, with amended No. 46/8 August 2025-rev). Given the retrospective, non-interventional design and the use of anonymized data, the requirement for informed consent was waived.

## 3. Results

All findings are diagnosis-level, institutional case-mix indicators derived from ICD-10 topography/behavior codes. ICD-10 provides a standardized, reproducible mapping of CMF sites (lip/oral cavity, salivary glands, sinonasal/ear, eye/adnexa, craniofacial bone/soft tissue/nerve, cutaneous head/neck) that enables year-over-year comparisons of the service’s recorded tumor burden. Because morphology (ICD-O) was not available, site-based entity classes are used as clinical proxies; results are not population incidences.

### 3.1. Frequency of CMF Tumor Diagnoses (2012–2016)

#### 3.1.1. Outpatient and Emergency Service Volumes

**Figure 1:**
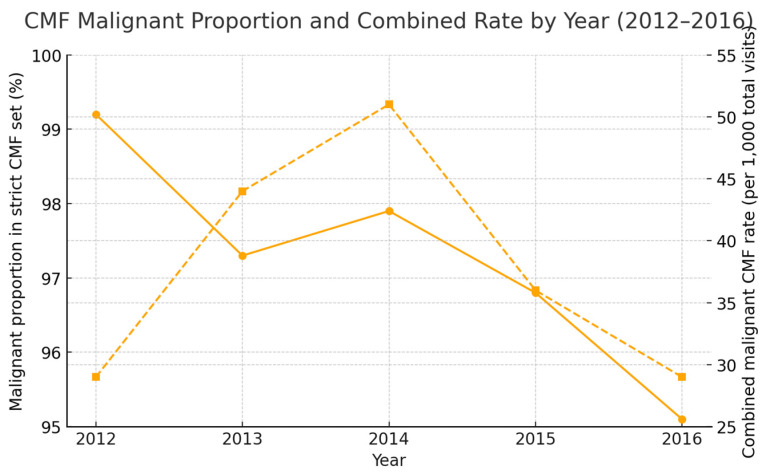
Strict CMF set (diagnosis-level) malignant proportion and combined malignant rate by year (2012–2016); (left y-axis: %, right y-axis: per 1000 total visits; x-axis: year).

Between 2012 and 2016, the department recorded 51,942 outpatient consultations and 20,789 ED consultations, including 1627 urgent outpatient visits and 13,746 urgent ED presentations. In parallel, within the outpatient stream, there were 11,959 tumor-related diagnoses (CMF and non-CMF) across the period (Table 1 and Figure 1). CMF diagnoses outnumbered non-CMF in 2012, 2013, and 2016, whereas non-CMF exceeded CMF in 2014–2015. Total outpatient tumor-related diagnoses decreased from 2013 (2852) to 2015 (1862) and remained low in 2016 (1816) relative to earlier years.

Most tumor-related diagnoses were CMF, with fewer non-CMF tumor diagnoses. A sharp contrast was observed in 2016, when the number of CMF diagnoses substantially exceeded non-CMF diagnoses.

Line plot showing (i) the proportion malignant within the strict CMF set (%) and (ii) the combined malignant rate per 1000 total visits by year. The solid line shows the proportion of malignant within the strict CMF set (diagnosis-level) (%); the dashed line shows the combined malignant rate per 1000 total visits. Strict CMF set (diagnosis-level) = all malignant CMF ICD-10 codes plus D00.0 (oral cavity CIS) and D14.1 (benign middle ear); unit of analysis = diagnosis level. The combined rate uses outpatient + ED visits in the denominator and excludes inpatient diagnoses from the numerator to preserve consistency. Values are institutional case-mix indicators, not population incidences.

#### 3.1.2. Malignant Share Within the CMF Case Mix (Primary Analysis)

In the primary analysis restricted to the strict CMF set (diagnosis-level) (malignant CMF diagnoses plus D00.0 and D14.1), the proportion malignant was 99.2%, 97.3%, 97.9%, 96.8%, and 95.1% in 2012–2016 (overall 97.4%). As an institution-level indicator, the combined malignant rate per 1000 total visits (outpatient + ED) was 30.6, 43.9, 52.6, 34.4, and 26.7 for 2012–2016, respectively; the numerator for this combined rate excludes inpatient diagnoses to match the outpatient+ED denominator.

Sensitivity Analysis (Denominator Choice). When broadening the denominator to include common benign CMF entities (D10.x, D16.4), the malignant proportion decreases markedly, especially in years with many benign diagnoses (e.g., 2016), illustrating that the strict metric functions as a case-mix monitor rather than a prevalence measure.

This single-center, five-year analysis provides institution-level evidence on cranio-maxillofacial (CMF) tumors in Western Romania, using standardized ICD-10 recoding and a diagnosis-level unit of analysis. To avoid misinterpretation, we emphasize that the key metric reported here is the proportion malignant within the strict CMF set (diagnosis level)—not a “malignancy rate” and not a population prevalence/incidence estimate.

Analytic frame and interpretation. By design, the strict CMF set (diagnosis-level) denominator includes all malignant CMF codes and only two non-malignant categories directly relevant to CMF workflows (D00.0 oral cavity CIS; D14.1 benign middle ear). This deliberate denominator makes most records in the set malignant; consequently, a malignant proportion in the 95–99% range is expected by construction. The result is therefore methodologically and arithmetically correct, but it should be interpreted as an institutional case-mix indicator, not as a measure of CMF burden in the population.

To guide readers, we clearly separate department-wide context metrics (Table 2 and Table 3)—calculated within total recorded conditions—from the strict CMF analysis. These context metrics reflect service mix and coding practices in a given year; they should not be interpreted as CMF prevalence/incidence and are not directly comparable to strict CMF set (diagnosis-level) proportions.

**Figure 2:**
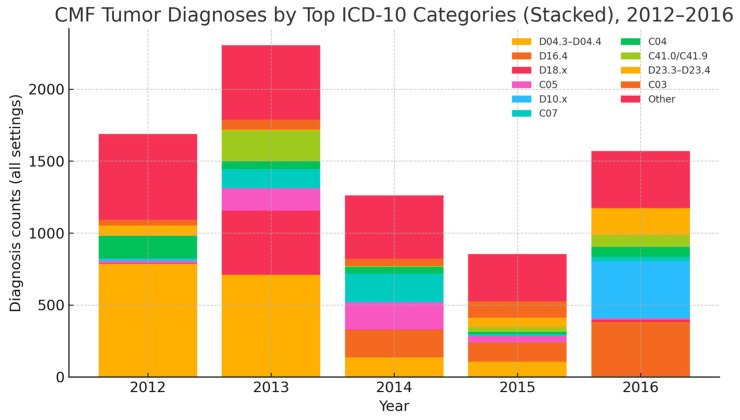
CMF tumor diagnoses by ICD-10 site category and year (stacked), all settings, 2012–2016. Stacked sums equal the total annual diagnoses (per year). Stacked bars display annual diagnosis counts across all settings (outpatient, ED, inpatient). Bars aggregate the top 10 ICD-10 categories by cumulative frequency; the remainder are grouped as Other. Wildcards follow ICD-10 notation (e.g., D18.x).

**Figure 3:**
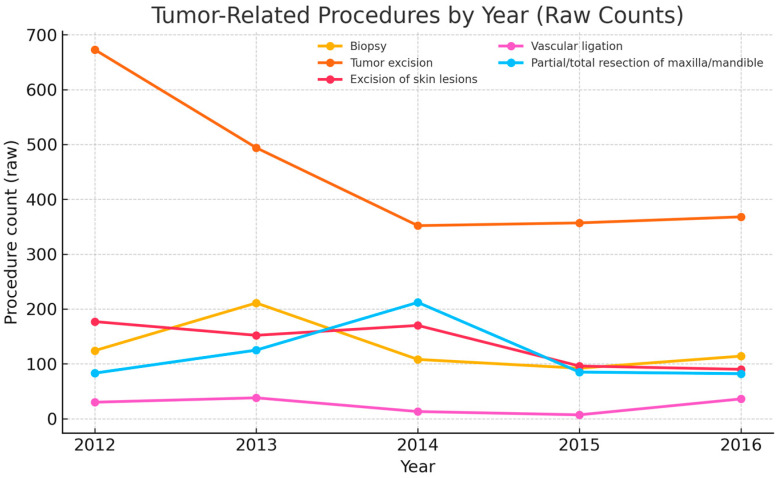
Tumor-related procedures by year and counts by year (2012–2016).

Five-year time-trend summary (2012–2016). Malignant CMF diagnoses totaled 2729, with annual counts of 519, 642, 751, 450, and 367 (peak 2014, subsequent decline). The combined malignant CMF rate per 1000 total visits (outpatient + ED) was 30.6, 43.9, 52.6, 34.4, and 26.7. The proportion malignant within the strict CMF set (diagnosis-level) was 99.2%, 97.3%, 97.9%, 96.8%, and 95.1%. Site distribution (Figure 2) shows oral/oropharyngeal and salivary entities as dominant classes, with coding-change signals for selected categories noted below. Procedure activity (Figure 3) is led by tumor excisions and biopsies, with craniofacial bone resections performed selectively; volumes contracted in 2015–2016.

#### 3.1.3. Department-Wide Context (Table 2 and Table 3; Not CMF Prevalence)

Table 2 and Table 3 report department-wide proportions calculated on all recorded conditions (“total recorded conditions”), not only CMF tumor diagnoses. These values reflect service mix (e.g., non-tumor presentations, inflammatory disease, trauma) and coding practices in a given year. They are therefore context-only indicators and should not be interpreted as CMF prevalence/incidence nor compared directly with strict CMF set (diagnosis-level) proportions. Percentages in Table 3 refer to tumor diagnoses only; the remaining share represents non-tumor conditions recorded in the department that year. The primary inference remains that, in Section 3.1.2, the malignant share within the strict CMF set (diagnosis-level) is very high (99.2 to 95.1%), with rates per 1000 visits peaking in 2014 and decreasing thereafter.

#### 3.1.4. Tumor Distribution by ICD-10 Site Category

Based on ICD-10 coding across all settings, the highest annual count of CMF tumor diagnoses occurred in 2013 (*n* = 2304) and the lowest in 2015 (*n* = 853) (Table 4). In 2016, benign neoplasms of the mouth and pharynx (D10.x) were most frequent (*n* = 396), followed by tongue (C02, *n* = 97) and floor of mouth (C04, *n* = 70); benign bone tumors (D16.4, *n* = 376) and benign skin lesions (D23.3–D23.4, *n* = 185) also increased in 2016 (see Table 2). Marked year-to-year variation for selected categories (e.g., C01, C07, C41.0/C41.9, D18.x, D04.3–D04.4) likely reflects coding/extraction differences across years and should be interpreted cautiously.

These ICD-10 site codes are the WHO standard for administrative clinical reporting and allow consistent CMF site attribution over time in the absence of morphology, thereby supporting service benchmarking, coding quality review, and resource planning at the institutional level.

By entity class, oral/oropharyngeal and salivary groups dominate malignant caseload; cutaneous head/neck and sinonasal/middle ear vary by year; craniofacial bone/soft-tissue/nerve and eye/adnexa contribute smaller but consistent shares; and C77.0/C82–C83 are recorded as head–neck presentations and not interpreted as primary CMF. These classes are ICD-10 site-based proxies (not morphology-confirmed), and odontogenic vs. non-odontogenic separation was not feasible.

Coding-change signal. Several categories showed implausible step changes (e.g., D04.3–D04.4; C07; C01; C41.0/C41.9; D18.x). Although consistent with coding/extraction artifacts, no formal audit trail was available to confirm the timing or nature of any EHR/query/codebook changes. These time series should therefore be interpreted cautiously and validated against pathology logs where possible.

In Table 3, percentages are out of all recorded conditions; non-tumor conditions account for the remaining share to 100%.

In addition to the primary strict CMF analysis, we report department-wide context indicators (Table 3 and Table 4) calculated as proportions within total recorded conditions; these are not used to infer CMF burden.

Unless stated otherwise, results are reported at the diagnosis level. Visit volumes in Table 5 serve as denominators for rate calculations and are not directly comparable to diagnosis counts.

#### 3.1.5. Trends in Emergency, Urgent Care, and Ambulatory Streams

Over 2012–2016, total ED consultations declined modestly, while the urgent-ED share remained high year to year (see Table 5). We use these volumes strictly as denominators for combined rates and do not interpret them as diagnosis counts.

### 3.2. Department Admissions (All Causes), Context-Only

The following summaries describe all department admissions (not de-duplicated by person) and are presented as context only; they are not used as numerators or denominators for CMF-specific rates/proportions.

#### 3.2.1. Sex-Based Distribution

Among hospital admissions with sex recorded (*n* = 9046), men (*n* = 4811; 53.8%) slightly outnumbered women (*n* = 4235; 46.2%).

#### 3.2.2. Age-Based Distribution

Most admissions involved adults; based on Table 6, adults accounted for 8351/9046 (92.3%), and children (<18 years) accounted for 695/9046 (7.7%). Detailed adult/child splits by residence are shown in Table 6.

#### 3.2.3. Urban vs. Rural Distribution

Admissions were predominantly urban, with 5748 urban (63.6%) vs. 3298 rural (36.4%) over 2012–2016 (urban/rural approximately 1.7:1) (Table 6).

### 3.3. Most Common Surgical Procedures for Tumor Treatment (2012–2016)

The most frequent procedures were tumor excisions (average ~450/year), biopsies (92–211/year), and excision of skin lesions (90–177/year). More complex operations—partial/total maxillary or mandibular resections and vascular ligations—were less common but essential for advanced disease (Table 7). Procedure counts are linked to the same encounter as the tumor diagnosis.

Annual procedure counts are plotted in Figure 3.

Multi-line plot showing annual raw counts for biopsy, tumor excision, excision of skin lesions, vascular ligation, and partial/total maxillary or mandibular resection. Counts are linked to the same encounter as the tumor diagnosis. “Tumor excision” includes soft-tissue/wide excisions and oncologic resections not captured under “partial/total maxillary/mandibular resection”; parotidectomies and neck dissections performed without bone resection are included in this bucket.

## 4. Discussion

Scope and Interpretation. All inferences are diagnosis-level, hospital-based case-mix indicators from ICD-10 site/behavior codes (not population incidences). Standardization and differentiation. We applied explicit inclusion/exclusion criteria, restricted to CMF topographies, and reported entity classes (oral/oropharyngeal, salivary, cutaneous head/neck, sinonasal/middle ear, eye/adnexa, craniofacial bone/soft tissue/nerve, nodal/lymphoid). Without ICD-O morphology, SCC and odontogenic vs. non-odontogenic splits cannot be confirmed at the record level. Five-year signal. Malignant diagnoses peaked in 2014, then declined; visit-normalized malignant rates tracked similarly.

Limitations and Implications. Single-center, ICD-10 site-based coding without morphology; incomplete histopathology linkage; no stage/treatment/outcomes; and potential coding/extraction drift. Within its scope, this study informs capacity planning, coding QA, and pathway design; future work should integrate ICD-O morphology, staging, and outcomes in multicenter registries.

In our data, this indicator peaked in 2014 and declined thereafter, a pattern that is more plausibly explained by year-to-year shifts in coding/extraction and service flows than by abrupt epidemiologic changes [14,15,16,18,19,20].

Five-year signal. Malignant CMF diagnoses totaled 2729, peaking in 2014 with a subsequent decline; the combined malignant rate per 1000 total visits tracked the same pattern. The proportion malignant within the strict-CMF set (malignant + D00.0 + D14.1) remained ≥95% annually by construction, functioning as a case-mix monitor sensitive to coding/service-mix shifts. Entity distributions were dominated by oral/oropharyngeal and salivary classes, with expected variability in cutaneous and sinonasal/middle-ear categories.

The demographic profile supports this interpretation. Men constituted 53.8% of admissions with sex recorded—substantially closer to parity than the historically male-predominant head and neck pattern (~2–3:1) reported in multi-setting reviews [14,21]. Likewise, the concentration of cases in younger to middle-aged adults contrasts with cohorts in which median age at diagnosis often approaches 60 years [18,21]. Together, these differences likely reflect referral behavior and local risk exposure rather than a fundamentally different disease biology [11,12,22].

Site distributions (oral cavity/oropharynx prominence) align with the established role of tobacco and alcohol as dominant drivers in Eastern Europe, and with the strong interaction between these exposures [14,21,22]. In contrast to many high-income settings where HPV-associated oropharyngeal cancer has risen, our configuration is more consistent with a lifestyle-risk signature [13,23]. From a care-pathway perspective, the procedural mix—biopsies and ablative excisions are the most frequent and complex, maxillary/mandibular resections are less common—tracks contemporary guideline-based management for resectable disease [19,24,25]. The contraction in recorded operative volume in 2015–2016 should be read cautiously, as it coincides with documentation fluctuations and may reflect capacity or reporting effects rather than a true change in demand [17,20]. The persistent urban–rural imbalance (approximately 1.7:1) also mirrors access gradients described in lower-resource settings and likely contributes to later presentation outside metropolitan areas [15,17,23].

Etiologic context helps situate these observations. In Eastern Europe, tobacco and alcohol remain the principal drivers of oral and pharyngeal cancers, with a strong synergistic effect when combined [14,21,22]. In contrast, high-income settings have seen a rising fraction of HPV-associated oropharyngeal cancers with distinct epidemiology and prognosis [13,23]. Although our dataset lacked risk-factor fields, the site distribution and age profile are more consistent with lifestyle-related risk than with an HPV-predominant pattern [13,14,19,26].

Surgical management mirrored contemporary standards: diagnostic biopsies and ablative tumor excisions were most frequent, while complex maxillary/mandibular resections and vascular ligations were less common but essential for advanced disease patterns concordant with guideline-based pathways and evidence favoring timely surgical control (with appropriate neck management) in resectable oral cavity cancer [3,19,24,25]. The contraction in recorded operative volume after 2014 should be interpreted cautiously; without rate adjustment and audit data, capacity constraints and documentation changes remain the most plausible explanations [17,20].

Data-quality signals—abrupt year-to-year swings for selected ICD-10 categories—underscore the need for standardized site-specific capture (ICD-10/ICD-O), internal code audits, and linkage to population registries to benchmark institutional trends against external denominators [9,16,18,20]. Hospital-based datasets like ours complement registry statistics by detailing case-mix and procedure volumes, but comparability hinges on transparent denominators and consistent coding across years [14,15,16,18,19,20,22].

Clinical Relevance. Our findings point to several practical priorities along the patient pathway. First, clinicians should maintain a high index of suspicion for early oral cavity lesions [21], particularly of the tongue and floor of mouth, in adults with recognized risk profiles, and ensure rapid access to biopsy so that time to diagnosis is minimized; timely resection with appropriate neck management remains central and is consistent with contemporary evidence and guidelines [19,24,27,28]. Second, the persistent urban–rural gradient argues for targeted outreach and strengthened referral support outside metropolitan areas, where delayed presentation is more likely [15,17,21].

Limitations. Single-center, retrospective design; ICD-10 site-based coding without morphology (no SCC confirmation; no clean odontogenic vs. non-odontogenic separation); incomplete and variably timed histopathology linkage; lack of stage, treatment, and outcomes; and potential coding/extraction artifacts over time. These constraints limit causal inference and generalizability but are transparent and consistent with an institutional case-mix objective. Histopathology linkage was incomplete and occurred at nonuniform intervals, and key clinical covariates—stage, histology, treatments, outcomes, and patient-level risk factors (tobacco, alcohol, HPV, occupational exposures)—were unavailable, limiting causal inference and generalizability beyond our setting [14,15,16,17,18,19,20,21]. Apparent shifts in 2015–2016 may reflect information-system or workforce changes rather than true epidemiology; without a formal audit trail, causation cannot be established [17,20].

Future Research. Several directions follow naturally from these observations. A prospective, multicenter CMF registry with standardized topography/behavior coding (ICD-10/ICD-O), stage, histology, treatments, and outcomes would enable robust benchmarking and longitudinal tracking. Parallel risk-factor surveillance (tobacco, alcohol, HPV) is needed to sharpen prevention strategies. Service-delivery studies should quantify diagnostic and treatment intervals—especially in rural catchments—and relate them to outcomes. Finally, routine reporting of per-setting rates alongside combined institutional indicators would help disentangle changes in case-mix from fluctuations in throughput [9,16,18,19,20,21,23,24].

Implications. Within its intended scope—institutional case-mix monitoring—this study provides standardized CMF site distributions, visit-normalized malignant indicators, and procedure trends that can inform capacity planning, coding QA, and pathway design. Future work should integrate ICD-O morphology, staging, and outcomes within multicenter registries to enable histology-resolved analyses (e.g., SCC) and odontogenic subtyping.

## 5. Conclusions

In this diagnosis-level, five-year series restricted to CMF sites, malignant tumors dominated the institutional case-mix every year, with a 2014 peak in the combined visit-normalized indicator followed by a decline that likely reflects coding and service-mix dynamics rather than true incidence change. Benign tumors comprised a smaller, steady component with a modest rise in 2016, consistent with detection/reporting effects. The near-parity sex ratio and younger-to-middle-aged profile—together with oral cavity/oropharyngeal predominance—are concordant with a lifestyle-risk signature typical of the region and contrast with HPV-driven patterns reported in many high-income settings. The urban–rural gap and the contemporaneous contraction in recorded procedures (2015–2016) are documented features of this caseload and should be considered when interpreting temporal trends. Overall, these institution-level observations support continued standardization of coding and denominators, cautious use of strict-set proportions as monitoring indicators, and closer alignment with population-based data to contextualize changes over time.

## Figures and Tables

**Table 1:** Outpatient consultations with tumor-related diagnoses (CMF, non-CMF, and total) by year; denominator: none (diagnosis counts at visit level).

Year	CMF Tumor Diagnoses	Non-CMF Tumor Diagnoses (Other Body Sites)	Total
2012	1698	930	2628
2013	2337	515	2852
2014	1304	1497	2801
2015	905	957	1862
2016	1725	91	1816
TOTAL	7969	3990	11,959

**Table 2:** Department-wide malignant share among all recorded conditions, 2012–2016 (context-only metric). Denominator = all conditions recorded in the department in the given year (not limited to CMF tumor diagnoses).

YEAR	Total Recorded Conditions	Malignant Tumor Diagnoses—CMF	Malignant Tumor Diagnoses—NON-CMF	Total Malignant Tumor Diagnoses	Benign Tumor Diagnoses
2012	13,368	1606	552	2158	470
2013	12,411	1813	412	2225	627
2014	12,772	1055	1030	2085	716
2015	8895	671	612	1283	579
2016	9120	580	76	656	1160

**Table 3:** Percentages are calculated out of all recorded conditions in the department for the given year. The benign and malignant columns show tumor diagnoses as a fraction of all conditions; the remainder, up to 100%, represents non-tumor conditions.

Year	Benign (N)	Benign (%)	Malignant (N)	Malignant (%)
2012	470	3.52	2158	16.14
2013	627	5.05	2225	17.93
2014	716	5.61	2085	16.32
2015	579	6.51	1283	14.42
2016	1160	12.72	656	7.19

**Table 4:** CMF tumor diagnoses by ICD-10 site category and year (2012–2016) across all settings; diagnosis counts.

ICD-10	ICD-10 DIAGNOSIS	2012	2013	2014	2015	2016
C00	Malignant neoplasm of lip	1	7	7	18	23
C01	Malignant neoplasm of base of tongue	1	1	4	107	16
C02	Malignant neoplasm of other/unspecified parts of tongue	40	1	2	5	97
C03	Malignant neoplasm of gum	41	72	51	114	4
C04	Malignant neoplasm of floor of mouth	157	53	47	17	70
C05	Malignant neoplasm of palate	15	155	190	46	10
C06	Malignant neoplasm of other/unspecified parts of mouth	103	8	10	29	29
C07	Malignant neoplasm of parotid gland	7	133	196	10	30
C08	Malignant neoplasm of other/unspecified major salivary glands	21	14	19	9	9
C09	Malignant neoplasm of tonsil	34	41	49	10	6
C10	Malignant neoplasm of oropharynx	28	44	43	1	6
C13	Malignant neoplasm of hypopharynx	7	21	20	2	1
C14	Other and ill-defined sites in the lip, oral cavity, and pharynx	22	5	19	27	13
C30.0/C30.1	Malignant neoplasm of nasal cavity and middle ear	39	70	1	33	10
C31	Malignant neoplasm of paranasal sinuses	2	13	41	2	3
C41.0	Malignant neoplasm of bones of skull and face	1	3	6	5	2
C41.0/C41.9	Malignant neoplasm of bone (skull/face and unspecified) *	1	213	5	34	83
C43.3–C43.4	Malignant melanoma of skin of face/scalp/neck	160	9	2	1	10
C44.3–C44.4	Other malignant neoplasms of skin of face/scalp/neck	2	59	19	18	53
C47.0	Malignant neoplasm of peripheral nerves of head/face/neck *	7	11	54	2	1
C49.0	Malignant neoplasm of connective/soft tissue of head/face/neck *	1	2	1	1	16
C69	Malignant neoplasm of eye and adnexa	1	4	52	20	40
C77.0	Secondary malignant neoplasm of lymph nodes of head/face/neck †	2	3	45	1	9
C82 (HN)	Follicular lymphoma (head/neck presentation) †	2	1	2	11	1
C83 (HN)	Diffuse non-Hodgkin lymphoma (head/neck presentation) †	7	1	5	1	4
D00.0	Carcinoma in situ of oral cavity	3	12	5	1	17
D02.0	Carcinoma in situ of middle ear *	2	8	10	4	1
D03.3–D03.4	Melanoma in situ of face/scalp/neck	106	110	10	4	1
D04.3–D04.4	Carcinoma in situ of skin of face/scalp/neck	785	708	137	107	5
D16.4	Benign neoplasm of bones of skull and face	1	2	191	131	376
D17.0	Benign lipomatous neoplasm of skin/subcutis of head/face/neck	1	61	2	1	25
D18.X	Hemangioma and lymphangioma (head/neck sites)	4	445	1	1	16
D23.3–D23.4	Benign neoplasm of skin of face/scalp/neck	71	6	1	65	185
D10.X	Benign neoplasm of mouth and pharynx *	11	2	3	1	396
D14.1	Benign neoplasm of middle ear *	1	6	11	14	2
TOTAL (INCLUDED ROWS)		1687	2304	1261	853	1570

Notes: Entries marked * are restricted to head/face/neck sites; † regional nodes (head/neck, C77.0); The large year-to-year swings flagged above likely reflect coding/extraction differences rather than true epidemiologic variation. C41.9 entries were included only when the EHR explicitly documented the head/face/neck site; otherwise, they were excluded as ambiguous.

**Table 5:** Annual clinical activity volumes by setting (2012–2016).

Year	Outpatient Total	Outpatient Urgent	ED Total	ED Urgent
2012	12,565	329	4399	2908
2013	10,422	402	4205	2775
2014	10,071	314	4209	2692
2015	9089	239	4004	2762
2016	9795	343	3972	2609
TOTAL	51,942	1627	20,789	13,746

Notes: “Urgent” is a subset of ‘Total’ for each setting; totals include urgent contacts.

**Table 6:** Cumulative monthly hospital admissions by residence (urban/rural) and age group, 2012–2016; denominator: none (admission counts).

Month	Total (Urban + Rural)	Urban Total	Urban Adults	Urban Children	Rural Total	Rural Adults	Rural Children
January	815	491	457	34	324	290	34
February	779	515	484	31	264	236	28
March	944	591	563	28	353	316	37
April	692	437	407	30	255	231	24
May	799	507	483	24	292	256	36
June	820	576	539	37	244	212	32
July	801	519	480	39	282	252	30
August	641	387	356	31	254	230	24
September	718	445	423	22	273	248	25
October	805	490	459	31	315	281	34
November	726	469	444	25	257	235	22
December	506	321	301	20	185	168	17
Total	9046	5748	5396	352	3298	2955	343

**Table 7:** Most common tumor-related surgical procedures (2012–2016).

Procedure	2012	2013	2014	2015	2016
Biopsy	124	211	108	92	114
Tumor excision	673	494	352	357	368
Excision of skin lesions	177	152	170	96	90
Vascular ligation	30	38	13	7	36
Partial/total resection of maxilla/mandible	83	125	212	85	82

Footnote: “Biopsy” includes needle/incisional/excisional biopsies of lymph nodes, salivary glands, soft tissue, bone, and skin performed in tumor work-ups. “Tumor excision” includes wide local excisions and ablative oncologic resections not involving bone resection and not coded separately as parotidectomy or neck dissection (these latter two were included under “tumor excision” in our OR labels when bone was not resected). “Partial/total resection of maxilla/mandible” captures segmental/hemimandibulectomy and partial/total maxillectomy. “Vascular ligation” includes ligation for tumor-related hemorrhage. Counts represent procedures linked to the same encounter as the tumor diagnosis.

## Data Availability

The data will be available from the corresponding authors upon reasonable request. De-identified aggregates are available upon reasonable request, subject to institutional and data-protection approvals.

## Abbreviations

The following abbreviations are used in this manuscript:

CMF	craniomaxillofacial
ED	emergency department
EHR	electronic health record
ICD-10	International Classification of Diseases, 10th Revision
WHO	World Health Organization
